# Signaling in the gas phase: comparative volatile profiling of biocontrol *Trichoderma* spp. and aflatoxin-producing *Aspergillus* spp. in isolation and co-culture using HS-SPME GC-MS

**DOI:** 10.3389/fmicb.2026.1873321

**Published:** 2026-10-09

**Authors:** Zilfa Irakoze, Nataliya M. Voloshchuk, Joshua D. Lambert, Helene Hopfer, Josephine Wee

**Affiliations:** 1 Department of Food Science, The Pennsylvania State University, University Park, PA, United States; 2 One Health Microbiome Center, Huck Institutes of the Life Sciences, The Pennsylvania State University, University Park, PA, United States

**Keywords:** biocontrol, microbial interactions, toxigenic *Aspergillus* spp., *Trichoderma*, untargeted metabolomics, volatile organic compounds (VOCs)

## Abstract

**Introduction:**

Food contamination by aflatoxin-producing *Aspergillus* spp. remains a major food safety and public health concern. *Trichoderma* spp. and their metabolites are promising biocontrol agents, yet the volatile organic compound (VOC)-mediated interactions underlying growth inhibition of *Aspergillus* spp. are not fully understood.

**Methods:**

We applied untargeted metabolomics using Headspace Solid Phase Microextraction Gas Chromatography–Mass Spectrometry (HS-SPME-GC-MS) and multivariate analyses to profile VOCs from three *Trichoderma* strains (*T. asperellum* BTU, *T. virens* OSK13, *T. harzianum* OSK34) in monoculture and in co-culture with *Aspergillus flavus* NRRL 3357 and *A. parasiticus* B62. Principal component analysis (PCA), ANOVA, PLS-DA, and Spearman correlation were used to characterize VOC profiles and identify discriminant compounds.

**Results:**

Co-cultures produced 30 VOCs across 9–12 chemical classes, dominated by sesquiterpenes, alcohols, and ketones. Among the *Trichoderma* strains, BTU (against *A. flavus* 3357) and OSK34 (against *A. parasiticus* B62) showed the greatest VOC abundance and diversity. PCA confirmed *Trichoderma*-driven changes in VOC profiles during co-cultures, with OSK13 and OSK34 sharing greater VOC profile similarity compared to BTU. In addition, *Trichoderma* spp. produced both species-specific and shared VOCs, with some also shared with *Aspergillus* spp. ANOVA further identified significant upregulation and downregulation of VOCs during co-culture. Finally, PLS-DA, ANOVA, and Spearman correlation identified four key discriminant VOCs: 6-pentyl-2H-pyran-2-one, 2-pentylfuran, 1-decanol, and 1-ethenyl-4-methoxybenzene. These VOCs were most abundant in BTU-*Aspergillus* co-cultures with observed antifungal activity.

**Discussion:**

These findings provide insight into potential mechanisms of *Aspergillus* growth inhibition by *Trichoderma* spp. through comparison of monocultures to co-cultures. In addition, the four identified key discriminant VOCs make promising VOC targets for selecting *Trichoderma* strains for biocontrol of toxigenic *Aspergillus*, particularly *A. flavus*. Together, this study establishes a metabolomic framework for VOC-mediated biocontrol and a basis for optimizing *Trichoderma* selection for biocontrol purposes. Future research should validate the bioactivity roles of these key VOCs and their effect on aflatoxin production, test their antifungal mechanisms, examine synergistic effects, and assess environmental influences to strengthen metabolomics-informed biocontrol strategies for reducing aflatoxin contamination in food systems.

## 1 Introduction

The increasing global population is projected to reach nearly 10 billion by 2050, necessitating a substantial increase in food production to meet demand (United Nations, 2023). Unfortunately, mycotoxins continue to impose a burden on food safety and nutrition security. Mycotoxins contaminate 60%–80% of agricultural crops, with about one quarter of these contaminations exceeding regulatory limits, leading to significant food loss and waste (Eskola et al., 2020). One of the most potent and concerning groups of mycotoxins is aflatoxins, a class of secondary metabolites primarily produced by *Aspergillus flavus* and *Aspergillus parasiticus*. There are four major aflatoxin types, B1, B2, G1, and G2, with aflatoxin B1 (AFB1) being the most toxic. Aflatoxins contaminate staple food crops such as maize, rice, and peanuts (Eskola et al., 2020). Human consumption of aflatoxin-contaminated food has been strongly linked to hepatocellular carcinoma, with elevated risks observed in populations co-infected with the Hepatitis B virus or Human Immunodeficiency Virus (Kew, 2003; Cardwell and Henry, 2004). Additional health consequences include impaired child growth, developmental delays, and immunosuppression (Gong et al., 2016).

Mycotoxin contamination is further compounded by environmental changes. Climate change is projected to intensify mycotoxin occurrence by creating conditions favorable for aflatoxin-producing fungi. Rising temperatures and shifting rainfall patterns are expected to expand the range and prevalence of *A. flavus* and *A. parasiticus*, ultimately increasing contamination risks and food safety concerns worldwide (Leggieri et al., 2021; Yu et al., 2022; Kos et al., 2023). These projections highlight the need for a resilient and environmentally safe solution to reduce mycotoxin contamination. In response, there has been growing interest in alternative biocontrol strategies that reduce reliance on synthetic chemical fungicides for managing plant pathogens. Synthetic fungicides face growing public resistance due to increased consumer concerns over health risks from residue transfer into food, rising microbial resistance, and environmental pollution (Tsitsigiannis et al., 2012; Ali et al., 2021; Weaver et al., 2022). Currently, the only available commercial biocontrol strains used for aflatoxin management are atoxigenic *A. flavus* formulations, such as AF36, Afla-Guard^®^, and various versions of Aflasafe^®^, which reduce toxigenic *Aspergillus* strains through competitive exclusion in the same ecological niche (Probst et al., 2011; Atehnkeng et al., 2014; Agbetiameh et al., 2019). However, reliance on a single biocontrol species is concerning, especially with climate change altering fungal ecology and increasing the spread of toxigenic strains. In addition, atoxigenic *A. flavus* strains are limited by their strict competitive mode of action, as well as potential recombination with toxigenic isolates, underscoring the need for complementary biocontrol strategies (Lewis et al., 2019).

Among the most promising biocontrol candidates are *Trichoderma* spp., which represent nearly 60% of registered biocontrol agents for plant health improvement (Guzmán-Guzmán et al., 2023). *Trichoderma* is a rapidly growing, widely distributed fungus that utilizes diverse nutrient sources, suppresses a broad range of plant pathogens, including *Aspergillus* spp., and enhances host plant growth, nutrient uptake, and resistance to abiotic stresses both *in vitro* and in the field (Glare et al., 2012; Mishra et al., 2016; Hu et al., 2020; Ferreira and Musumeci, 2021). Although *Trichoderma* spp. have not yet been commercialized for aflatoxin management, multiple studies have demonstrated effective inhibition of both the growth of toxigenic *Aspergillus* strains and aflatoxin biosynthesis in the laboratory and in the field (Mostafa et al., 2013; Sivparsad and Laing, 2016; Kifle et al., 2017; Ren et al., 2020; Peles et al., 2021). The biocontrol potential of *Trichoderma* spp. is linked to the multifaceted mechanisms, including competitive exclusion, mycoparasitism, and production of antagonistic secondary metabolites (antibiosis), known as volatile organic compounds (VOCs) and non-VOCs.

Volatile organic compounds (VOCs) are carbon-containing low molecular weight compounds with high vapor pressure. These characteristics enable their high volatility, which, combined with lower toxicity, makes them increasingly interesting for postharvest biocontrol (Boukaew et al., 2024). Our group and others have demonstrated that VOCs produced by *Trichoderma* spp. can effectively control fungal growth and toxin production by toxigenic *Aspergillus* spp. Voloshchuk et al. (2024) used a sandwiched plate assay to demonstrate that VOCs from *T. harzianum* (OSK-34) and *T. asperellum* (BTU) inhibited the growth of *A. flavus* by approximately 21% and *A. parasiticus* by 34%–44%. Additionally, *T. asperelloides* VOCs suppressed mycelial growth of both *A. parasiticus* and *A. flavus* by around 75% and 71%, respectively (Boukaew et al., 2024). However, fungal growth inhibition and aflatoxin suppression do not always co-occur. *Trichoderma* VOCs can inhibit *Aspergillus* growth without reducing toxin production and vice versa. This highlights the need to dissect the mechanisms underlying VOC-mediated interactions (Ren et al., 2022; Voloshchuk et al., 2024). Despite extensive interest in the use of *Trichoderma* as a biocontrol agent, there remains limited knowledge of the specific mechanisms underlying its ability to suppress *Aspergillus* growth and aflatoxin production. This knowledge gap is partly because relatively few studies have been conducted, and these have (1) characterized the VOC profiles of a limited number of *Trichoderma* spp., and (2) often focused on *Trichoderma* grown in monoculture or in co-culture with plant pathogens other than toxigenic *Aspergillus* (Ruangwong et al., 2021; Stoppacher et al., 2010). When evaluated in monoculture, *Trichoderma* strains have been shown to release VOCs belonging to diverse chemical classes such as alcohols, ketones, sesquiterpenes, and lactones, many of which are known to possess antifungal properties (Boukaew et al., 2024; Stoppacher et al., 2010). Nonetheless, no study to date has comprehensively examined how VOC production by *Trichoderma* is modulated during co-culture with toxigenic *Aspergillus* spp.

This study employs untargeted metabolomics, integrating Headspace Solid Phase Microextraction Gas Chromatography–Mass Spectrometry (HS SPME GC-MS) and multivariate statistical analyses, to examine how the most potent *Trichoderma* strains alter their VOC profiles during co-culture with toxigenic *Aspergillus* spp., linking these changes to growth inhibition of *Aspergillus* spp. We hypothesized that (1) co-cultures of the most inhibitory *Trichoderma* strains and toxigenic *Aspergillus* spp. would exhibit the greatest diversity and abundance of VOCs, contributing to stronger growth inhibition; (2) the volatile profiles of these co-cultures would be predominantly driven by *Trichoderma;* and (3) the transition from monoculture to co-culture would result in both conserved and species-specific shifts in VOC compositions. Collectively, the findings of this study offer new insights into the metabolomic pathways and regulatory VOCs involved in the antifungal activity of *Trichoderma*, highlighting the role of signaling in the gas phase as a key mechanism in microbial interactions. Understanding these interactions provides a foundation for optimizing *Trichoderma*-based biocontrol strategies and developing complementary approaches to mitigate toxigenic *Aspergillus* growth in food crops.

## 2 Materials and methods

### 2.1 Fungal strains and culture conditions

Two *Trichoderma* strains used in this study were a gift from Dr. Seogchan Kang (Penn State): *T. virens* (OSK13), and *T. harzianum* (OSK34), and one from BTU Biotech company (Ukraine), *Trichoderma asperellum* (BTU). *Aspergillus parasiticus* B62 (ApB62), was a gift from Dr. John Linz (Michigan State University) and *Aspergillus flavus* NRRL 3357 (A.f. 3357) was from the Agricultural Research Service (ARS) Culture Collection (Peoria, IL, USA). A.f. 3357 is a reference strain known for high aflatoxin production, while ApB62 is a mutant derived from the high-producing parent strain SU-1 (ATCC 56775). ApB62 was genetically modified through a single amino acid substitution in *nor-1* resulting in a mutant carrying a single amino acid substitution of a non-functional Nor-1. This mutant accumulates norsolorinic acid, an orange-colored precursor of aflatoxin biosynthesis, enabling fast visual detection of aflatoxin inhibition when screening multiple *Trichoderma* strains (Wee et al., 2016). All fungal strains were previously identified and validated using ITS and Tef1 primers (Voloshchuk et al., 2024). Fungal strains were cultured on potato dextrose agar (PDA; Difco™, Becton, Dickinson and Company, Franklin Lakes, NJ, USA) at 30 °C for 5 days. Spores were then harvested using 0.05% Tween-20 in sterile distilled water, filtered in a cell strainer, quantified using a hemocytometer, and stored as stocks in 25% glycerol at −80 °C.

### 2.2 Preparation of headspace vials for dual-culture VOC profiling

The experimental setup for automated solid phase microextraction (SPME) sampling of VOCs was adapted from Yalage Don et al. (2020) with modifications. Briefly, 2 mL of PDA was dispensed onto one side of a 20 mL headspace glass vial (Restek Corporation, Bellefonte, PA, USA) and left to solidify horizontally, creating a solid agar surface restricted to one side of the vial. After solidification, an additional 2 mL of PDA was poured into the opposite side of the vial, ensuring no contact between the two agar sections. Vials were left to solidify for 1 h at 25 °C prior to fungal inoculation. For fungal inoculation, each *Aspergillus* strain (ApB62 or A.f. 3357) was center inoculated on one side of the agar using 1 μL of a spore suspension containing 10^5^ spores/mL. This volume and concentration were selected based on a preliminary study conducted to monitor *Trichoderma* growth and prevent overgrowth, maintaining no contact with *Aspergillus* spp. over 3 days of co-culture. On the opposite side of the agar, 1 μL of spore suspension (10^5^ spores/mL) from one of the selected biocontrol *Trichoderma* spp. (OSK34, OSK13, or BTU) was point-inoculated. These strains were chosen based on previously reported high VOC-driven antifungal activity against both *Aspergillus* spp. in a sandwich plate assay (Voloshchuk et al., 2024). For each *Trichoderma-Aspergillus* combination, corresponding monocultures and non-inoculated PDA control were included to control for background volatiles, as summarized in Table 1. All experimental and control vials were prepared in triplicate. Following inoculation, vials were sealed with screw caps fitted with PTFE/silicone septa (Restek Corporation) and incubated horizontally at 30 °C for 72 h to allow fungal interaction and VOC accumulation.

**TABLE 1:** Summary of *Trichoderma-Aspergillus* combinations and controls evaluated in this study.

Pathogen *(Aspergillus)*	Biocontrol (*Trichoderma)*	Culture type	Percent growth inhibition (%)
A.f. 3357	–	Monoculture	–
ApB62	–	Monoculture	–
–	BTU *(T. asperellum)*	Monoculture	–
–	OSK13 *(T. virens)*	Monoculture	–
–	OSK34 *(T. harzianum)*	Monoculture	–
A.f. 3357	BTU *(T. asperellum)*	Co-culture	31.8^b^
A.f. 3357	OSK13 *(T. virens)*	Co-culture	21.2^a^
A.f. 3357	OSK34 *(T. harzianum)*	Co-culture	22.7^ab^
ApB62	BTU *(T. asperellum)*	Co-culture	41.2^a^
ApB62	OSK13 *(T. virens)*	Co-culture	35.3^a^
ApB62	OSK34 *(T. harzianum)*	Co-culture	44.1^a^
–	–	PDA Control	–

Percent growth inhibition values reported in this table were derived from (Voloshchuk et al., 2024). Within each *Aspergillus* strain, co-culture growth inhibitions with different lowercase superscript letters are significantly different (*p* < 0.05), based on one-way ANOVA followed by Tukey’s HSD test.

### 2.3 HS-SPME gas chromatography and mass spectrometry analysis

Headspace VOCs produced in both fungal co-culture and monoculture vials were analyzed using headspace solid-phase microextraction (HS-SPME) coupled with gas chromatography–mass spectrometry (GC–MS). Just before HS-SPME analysis, an internal standard (IS), which comprised 1 μL of methanolic D8-naphthalene (Sigma-Aldrich, St. Louis, MO, USA) solution, was introduced directly into the headspace of each vial by piercing the septum with a glass syringe (Agilent Technologies, Santa Clara, CA, USA) without opening the vial. The IS concentration for A.f. 3357 (245 ng/mL) and ApB62 (98 ng/mL) was determined through preliminary optimization to ensure that the peak intensity of the IS was proportional to the dominant VOCs in each treatment.

To enhance VOC analysis from fungal cultures, key parameters of the SPME sampling process were systematically optimized and detailed here. First, two SPME fibers were evaluated: polydimethylsiloxane/divinylbenzene (PDMS/DVB) and polydimethylsiloxane/carboxen/divinylbenzene (PDMS/CAR/DVB), both 20 mm in length (Supelco^®^, Bellefonte, PA, USA). Each fiber (pre-conditioned at 250 °C for 30 min before use) was mounted on a Gerstel MPS robotic autosampler (Gerstel, Mülheim an der Ruhr, Germany) and tested under identical conditions to compare the diversity and relative abundance of VOCs detected. While both fibers yielded comparable overall VOC profiles, PDMS/DVB was selected for subsequent analyses due to its superior resolution of compounds eluting around 13 min (Supplementary Figure 1).

Second, headspace sample incubation and extraction times were optimized using a full factorial design to maximize VOC diversity and abundance while minimizing relative standard deviation across replicates. Holding incubation and extraction temperature constant at 35 °C, incubation and fiber exposure times were varied between 1 and 30 min. The combination of 15 min incubation and 30 min of extraction yielded the highest metabolite diversity and abundance with minimal variability and was therefore selected for all subsequent analyses.

All GC–MS analyses were performed on an Agilent 7890B GC system (Agilent Technologies, Santa Clara, CA, USA) coupled with an Agilent 5977B MSD (Agilent Technologies). The two *Aspergillus*-*Trichoderma* interaction analyses were run independently, and in each system, the samples were run in a randomized treatment order. Empty vials were used to control for contamination from the vial, septa, and instrument. SPME fibers were thermally cleaned for 10 min at 250 °C to control for carryover. Samples were analyzed in randomized treatment order, and replicates of the same treatment were spread out to account for potential order effects. Thermal desorption of VOCs was conducted into a SPME inlet liner (Restek Corporation) with the inlet operated in splitless mode for 1.2 min, and held at a temperature of 250°C. Ultra-pure Helium (99.999%, Linde Gas & Equipment Inc., State College, PA, USA) was used as the carrier gas at a constant flow of 1.2 mL/min. VOCs were separated on a StabilWax-MS capillary column (30 m × 250 μm ID × 0.25 μm df; Restek Corporation). The oven program started at 35 °C (2 min hold), ramped at 10 °C/min to 250 °C, with a final hold at 250 °C for 3 min. The MSD transfer line was held at 250 °C, the ion source at 230 °C, and the quadrupole at 150 °C. Data were acquired in electron ionization (EI) mode at 70 eV in full scanning mode (m/z range of 32–350, 8 scans/s). Retention indices (RI) of identified compounds were calculated using an alkane standard mix (C5-C40; Supelco^®^, Bellefonte, PA, USA) that was analyzed with the same parameters.

### 2.4 Data processing, peak identification, metabolomic profiling

Chromatogram files were converted to .cdf format using Agilent MSD Chemstation (version F.01.03.2357) for peak detection, alignment, and deconvolution using PARADISE (Johnsen et al., 2017). Aligned peaks were annotated based on mass spectral matching using the NIST-14 library (National Institute of Standards and Technology [NIST], 2014). We excluded VOCs that appeared in PDA-only controls at levels above 10% of the maximum found in treatment samples to remove media-derived compounds. Remaining VOCs were further validated by comparing their RI with entries in the NIST Chemistry WebBook. For VOCs with commercial standards in our possession, RI and mass spectra were confirmed under the same GC-MS conditions (Supplementary Table 1). Pure analytical standards were obtained from Thermo Fisher Scientific (Waltham, MA, USA), Tokyo Chemical Industries (TCI America, Portland, OR, USA), and Sigma-Aldrich (St. Louis, MO, USA) (Supplementary Table 1). Only compounds that were not from media with a validated RI, spectral match above 70%, or confirmed with a standard were retained for further analysis (Full list of VOCs before the 10% threshold, Spectral match and RI filtering uploaded on ScholarSphere (Irakoze et al., 2026). All identified VOC peak areas were normalized to the IS by dividing each VOC’s peak area by that of the IS and multiplying by the known IS concentration. The semi-quantitative IS-normalized concentrations (pg/vial) were used for all subsequent analyses.

### 2.5 Statistical analyses

To compare VOC abundance and diversity across treatments, the identified VOCs were classified into chemical classes using PubChem and the literature. Summed concentrations of VOCs in each class were visualized as stacked bar plots in RStudio version 2024.09.0+375 (Posit team, 2024). The dataset containing VOCs and their IS-normalized concentrations for each pathogen-*Trichoderma* was independently analyzed in MetaboAnalyst 6.0 (Pang et al., 2024). After integrity checks, data were log_10_-transformed and mean-centered to improve comparability across treatments. Principal Component Analysis (PCA) was performed to assess clustering of *Trichoderma* and *Aspergillus* in monocultures and in co-cultures. To evaluate the effects of *Trichoderma-Aspergillus* interactions on each VOC, one-way ANOVA with false discovery rate (FDR) correction, followed by Tukey’s HSD *post hoc* testing, was conducted in RStudio version 2024.09.0+375 (Posit team, 2024). For all significantly different VOCs (*p* < 0.05), hierarchical clustered heatmaps of VOCs (rows) based on Euclidean distance and complete linkage were generated to visualize VOC abundance patterns across samples (columns). Partial Least Squares-Discriminant Analysis (PLS-DA) identified VOCs discriminating all *Trichoderma* co-cultures, with model validation by leave-one-out cross-validation retaining two components (Q^2^ = 0.93, R^2^ = 0.97 and Q^2^ = 0.93, R^2^ = 0.99 for A.f. 3357 and ApB62, respectively). VOCs with VIP ≥ 1.0 were considered putative key discriminants. To increase confidence in putative bioactive VOC detection, correlation analysis of VOC abundance and percent growth inhibition of the different *Trichoderma* strains in co-cultures was conducted. The IS-normalized VOC concentrations were averaged across replicates (*n* = 3) for each *Trichoderma* spp. co-culture and *Aspergillus* monoculture before correlation analysis. Spearman’s rank correlations were calculated between mean VOC levels and previously reported mean percent growth inhibition (Voloshchuk et al., 2024) to find potential bioactive VOCs associated with percent growth inhibition (r_s_ ≥ 0.5, *p* < 0.05). Due to the small sample-to-variable ratio of the data, the PLS-DA model and Spearman correlation were used for ranking rather than predictive classification of VOCs. All statistical analyses were conducted independently for each *Aspergillus* spp., and the consensus VOCs were highlighted as putative antifungal VOCs based on high ranking by the multiple statistical analyses.

## 3 Results and discussion

### 3.1 The greatest VOC diversity and abundance were observed in the most inhibitory *Trichoderma* strains in co-cultures with *Aspergillus* spp.

Co-culturing of *Trichoderma* strains with A.f. 3357 and ApB62 resulted in distinct patterns of VOC abundance and distribution of chemical classes. Across all co-cultures, comprehensive profiling identified 30 VOCs per pathogen interaction (Tables 2, 3), grouped into 12 functional compound classes for A.f. 3357 and 9 for ApB62. In both cases, sesquiterpenes dominated (8 and 7 compounds, respectively), followed by alcohols (6 each) and ketones (4 each). Other classes included carboxylic acids, esters, aromatic hydrocarbons, furan derivatives, hydroxyketones, lactones, monoterpenes, monoterpenoid ketones, and sesquiterpenoid alcohol. These findings align with previous studies showing that *Trichoderma* VOCs are chemically diverse and rich in sesquiterpenes, alcohols, and ketones, a diversity that could explain their antibiosis activity against a range of pathogens (Stoppacher et al., 2010; Shahiri et al., 2016).

**TABLE 2:** VOCs released by the interaction of the three *Trichoderma* species with A.f. 3357.

VOC number	Tentative annotation	Class	CAS number	RT	RI (observed)	Kováts RI	Confirmation/ identification method
AflaVOC1	Ethanol	Alcohol	64-17-5	2.83	–		MS
AflaVOC2	2-methyl-1-propanol	Alcohol	78-83-1	4.52	1103	1098	MS+RI+Standard
AflaVOC3	β-Phellandrene	Monoterpene	555-10-2	5.91	1208	1200	RI
AflaVOC4	2-methyl-1-butanol	Alcohol	137-32-6	5.96	1211	1211	RI + Standard
AflaVOC5	3-methyl-1-butanol	Alcohol	123-51-3	5.98	1213	1211	MS+RI+Standard
AflaVOC6	2-pentylfuran	Furan derivative	3777-69-3	6.25	1233	1235	MS+RI+Standard
AflaVOC7	3-Octanone	Ketone	106-68-3	6.62	1260	1262	MS+RI+Standard
AflaVOC8	Styrene	Aromatic hydrocarbon	100-42-5	6.69	1266	1262	RI +Standard
AflaVOC9	2-Octanone	Ketone	111-13-7	7.05	1292	1297	MS+RI+Standard
AflaVOC10	Acetoin	Hydroxyketone	513-86-0	7.15	1299	1290	MS+RI+Standard
AflaVOC11	3-Heptanone, 5-ethyl-4-methyl-	Ketone	27607-63-2	7.21	1304		MS
AflaVOC12	2-Pentenoic acid, 4,4- dimethyl-, methyl ester	Carboxylic acid ester	20607-85-6	7.50	1326		MS
AflaVOC13	2-n-Heptylfuran	Furan derivative	3777-71-7	8.96	1437		MS
AflaVOC14	Acetic acid	Carboxylic acid	64-19-7	9.29	1463	1463	MS+RI+Standard
AflaVOC15	Propanoic acid, 2-methyl-	Carboxylic acid	79-31-2	10.73	1578	1571	MS+RI
AflaVOC16	trans-α-Bergamotene	Sesquiterpene	13474-59-4	10.91	1592	1590	RI
AflaVOC17	α-Guaiene	Sesquiterpene	13474-59-5	11.02	1601	1596	RI
AflaVOC18	Cyclohexane, 1-ethenyl-1-methyl-2,4-bis(1-methylethenyl)-, [1S-(1α,2β,4β)]-	Sesquiterpene	515-13-9	11.03	1602	1600	RI
AflaVOC19	2-Undecanone	Ketone	112-12-9	11.09	1608	1606	MS+RI
AflaVOC20	Caryophyllene	Sesquiterpene	87-44-5	11.12	1610	1612	RI +Standard
AflaVOC21	1-Decanol	Alcohol	112-30-1	12.36	1716	1714	RI
AflaVOC22	Germacrene D	Sesquiterpene	23986-74-5	12.47	1726	1726	RI
AflaVOC23	Naphthalene, 1,2,3,4,4a,5,6,8a-octahydro-7-methyl-4-methylene-1-(1-methylethyl)-, (1α,4aβ,8aα)-	Sesquiterpene	39029-41-9	12.58	1736	1738	RI
AflaVOC24	β-Bisabolene	Sesquiterpene	495-61-4	12.59	1736	1730	RI
AflaVOC25	2-Butanone, 4-(2,6,6-trimethyl-2-cyclohexen-1-yl)-	Monoterpenoid ketone	31499-72-6	13.67	1834		MS
AflaVOC26	Naphthalene, 1,2,3,4-tetrahydro-1,6-dimethyl-4-(1-methylethyl)-, (1S-cis)-	Sesquiterpene	483-77-2	13.84	1849	1839	RI
AflaVOC27	Phenylethyl alcohol	Alcohol	60-12-8	14.67	1929	1929	MS+RI+Standard
AflaVOC28	6-pentyl-2H-pyran-2-one	Lactone	27593-23-3	17.46	–		Standard
AflaVOC29	Ethyl hexadecanoate	Carboxylic acid ester	628-97-7	17.88	–		MS +Standard
AflaVOC30	Spiro[4.5]dec-8-en-7-ol, 4,8-dimethyl-1-(1-methylethyl)-	Sesquiterpenoid alcohol	61050-89-3	18.38	–		MS

MS (match probability > 70%), RI (retention index) using literature compiled in NIST Chemistry WebBook.

**TABLE 3:** VOCs released by the interaction of the three *Trichoderma* species with ApB62.

VOC number	Tentative annotation	Class	CAS number	RT	RI (observed)	Kováts RI	Confirmation/ identification method
AparVOC1	Ethanol	Alcohol	64-17-5	2.83	–		MS
AparVOC2	Propanoic acid, 2- methyl-, ethyl ester	Carboxylic acid ester	97-62-1	3.11	–		MS + Standard
AparVOC3	Butanoic acid, 2- methyl-, ethyl ester	Carboxylic acid ester	7452-79-1	3.99	1057	–	MS + Standard
AparVOC4	2-Methyl-1-propanol	Alcohol	78-83-1	4.52	1105	1098	MS+ RI+ Standard
AparVOC5	β-Phellandrene	Monoterpene	555-10-2	5.71	1195	1194	RI
AparVOC6	2-methyl-1-butanol	Alcohol	137-32-6	5.96	1213	1211	MS+ RI+ Standard
AparVOC7	3-methyl-1-butanol	Alcohol	123-51-3	5.97	1214	1211	MS+ RI+ Standard
AparVOC8	2-pentylfuran	Furan derivative	3777-69-3	6.25	1234	1235	MS+ RI+ Standard
AparVOC9	Ethyl hexanoate	Carboxylic acid ester	123-66-0	6.29	1237	1240	MS+ RI+ Standard
AparVOC10	Styrene	Aromatic hydrocarbon	100-42-5	6.61	1261	1262	RI + Standard
AparVOC11	3-Octanone	Ketone	106-68-3	6.62	1262	1262	MS+ RI+ Standard
AparVOC12	2-Octanone	Ketone	111-13-7	7.06	1293	1297	MS+ RI+ Standard
AparVOC13	3-Heptanone, 5-ethyl-4-methyl-	Ketone	27607-63-2	7.11	1297	–	MS
AparVOC14	Acetoin	Hydroxyketone	513-86-0	7.14	1299	–	MS + Standard
AparVOC15	2-Pentenoic acid, 4,4- dimethyl-, methyl ester	Carboxylic acid ester	20607-85-6	7.51	1327	–	MS
AparVOC16	3-Isopropyl-6,8a-dimethyl-1,2,4,5,8,8a-hexahydroazulene	Sesquiterpene	16661-00-0	9.71	1495	1495	RI
AparVOC17	trans-α-bergamotene	Sesquiterpene	13474-59-4	10.84	1587	1590	RI
AparVOC18	α-Guaiene	Sesquiterpene	13474-59-5	10.93	1594	1596	RI
AparVOC19	2-Undecanone	Ketone	112-12-9	11.09	1608	1606	MS +RI
AparVOC20	Butanoic acid, 2-methyl-	Carboxylic acid	116-53-0	11.95	1681	1682	MS+ RI+ Standard
AparVOC21	1-Ethenyl-4-methoxybenzene	Aromatic hydrocarbon	637-69-4	12.12	1696		MS
AparVOC22	1-Decanol	Alcohol	112-30-1	12.37	1717	1714	RI
AparVOC23	Germacrene D	Sesquiterpene	23986-74-5	12.46	1726	1726	RI
AparVOC24	1,3-Cyclohexadiene, 5-(1,5-dimethyl-4-hexenyl)-2- methyl-, [S-(R*,S*)]-	Sesquiterpene	495-60-3	12.47	1726	1726	RI
AparVOC25	Naphthalene, 1,2,3,4,4a,5,6,8a-octahydro-7-methyl-4-methylene-1-(1-methylethyl)-, (1α,4aβ,8aα)-	Sesquiterpene	39029-41-9	12.59	1737	1738	RI
AparVOC26	Cyclohexene, 3-(1,5-dimethyl-4-hexenyl)-6- methylene-, [S-(R*,S*)]-	Sesquiterpene	20307-83-9	13.04	1777	1775	MS +RI
AparVOC27	Benzene, 1-(1,5-dimethyl-4-hexenyl)-4-methyl-	Aromatic hydrocarbon	644-30-4	13.048	1778	1777	RI
AparVOC28	Phenylethyl alcohol	Alcohol	60-12-8	14.68	1931	1929	MS+ RI+ Standard
AparVOC29	6-pentyl-2H-pyran-2-one	Lactone	27593-23-3	17.42	–	–	MS+ Standard
AparVOC30	Spiro[4.5]dec-8-en-7-ol, 4,8-dimethyl-1-(1-methylethyl)-	Sesquiterpenoid alcohol	61050-89-3	18.34	–	–	MS

*MS (match probability > 70%), RI (retention index) using literature compiled in NIST Chemistry WebBook.

Strain-specific differences demonstrate that co-cultures involving *Trichoderma* strains BTU against A.f. 3357 and OSK34 against ApB62 exhibited greater VOC abundance and diversity. Against A.f. 3357, the BTU–Af3357 co-culture produced the highest total VOC abundance (pg/vial) and class diversity (Figure 1A); interestingly, BTU also had the highest percent growth inhibition (Table 1). Similarly, in ApB62 interactions, OSK34–ApB62, the strain with the highest growth inhibition (not statistically significant), displayed the highest VOC abundance and diversity (Figure 1B). These results suggest that enhanced VOC production may contribute to stronger antagonistic activity, a pattern that has also been observed in other studies, where *Trichoderma* isolates with higher VOC abundance/diversity showed greater inhibition of fungal pathogens such as *Rhizoctonia solani* (van Zijll de Jong et al., 2023). The elevated VOC abundance and diversity such as that observed between BTU and A.f. 3357, likely stem from an enhancement of secondary metabolism in response to pathogen detection through signaling in the gas phase (Li et al., 2018; Thomas et al., 2025). For example, the exposure of *T. virens* to *Fusarium oxysporum* VOCs led to a significant induction of the terpene synthase encoding gene *tps*, which could signify a potential upregulation of VOC production, such as terpenes, in exposure to pathogen VOCs (Li et al., 2018). VOC profile comparison between *Trichoderma* mono- and co-cultures is one approach to reveal pathogen-induced VOC changes, and give insights into the metabolomic mechanisms driving *Trichoderma* biocontrol against toxigenic *Aspergillus* spp.

**FIGURE 1:**
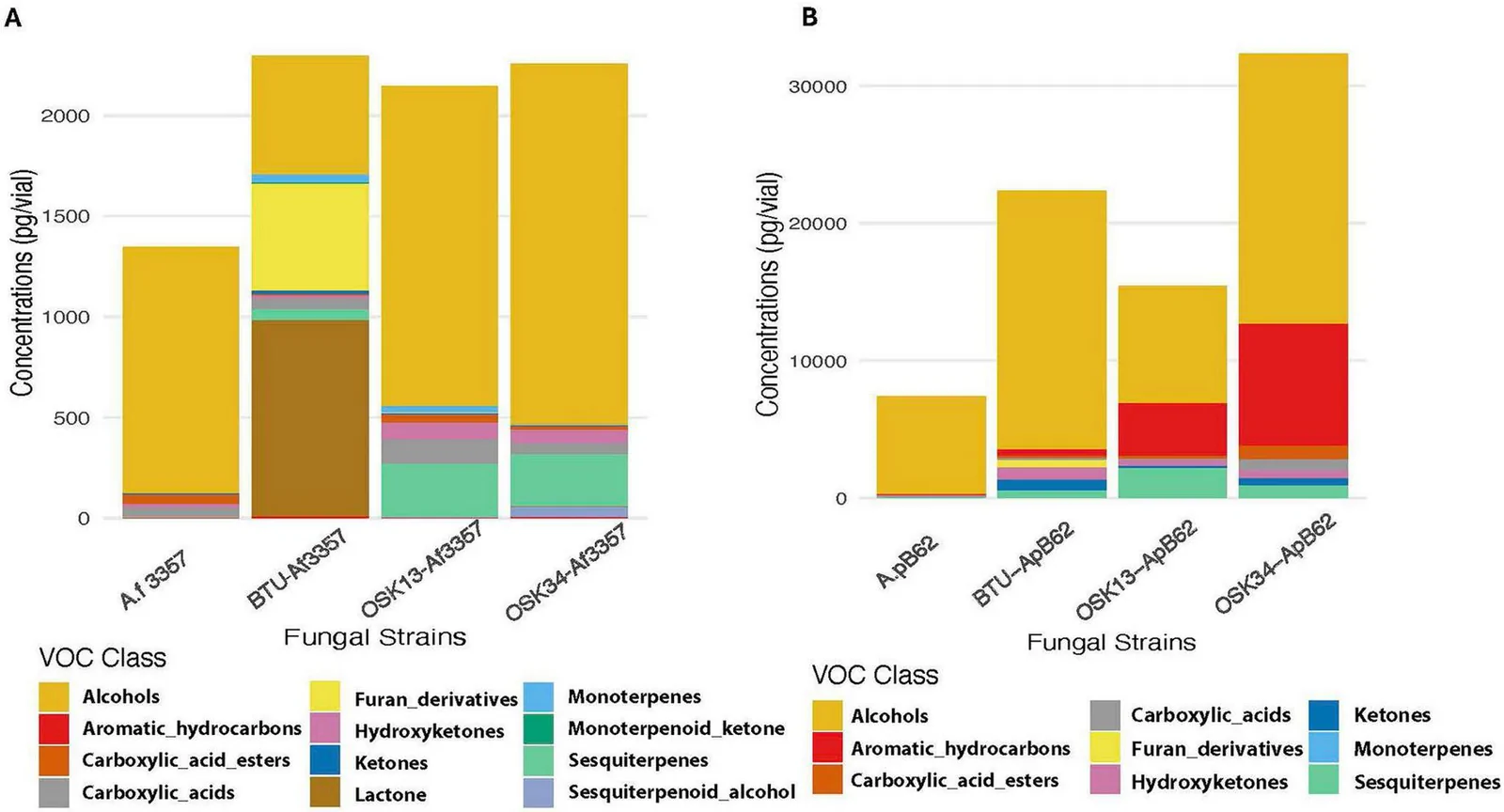
Class-level distribution of VOCs produced by *Aspergillus* in monoculture and its co-cultures with *Trichoderma* spp. VOC composition of panel **(A)** *A. flavus* NRRL 3357 (A.f. 3357) and **(B)** *A. parasiticus B62* (ApB62) and their corresponding co-cultures with the three evaluated *Trichoderma* spp. Stacked bars represent the sum of IS-normalized concentrations for all VOC classes detected in each treatment. Different colors represent distinct VOC classes.

### 3.2 Distinct VOC profiles across *Trichoderma* spp. drive VOC changes in co-cultures

To evaluate similarities and differences in volatile profiles between *Trichoderma* monocultures and co-cultures, a PCA was conducted. The first two principal components explained 74.9% of the variance for A.f. 3357 interactions (Figure 2A) and 57.5% for ApB62 interactions (Figure 2B). The score plots (Figure 2) show clear separation between *Trichoderma* strains and *Aspergillus* spp., with co-culture profiles clustered more closely with monoculture *Trichoderma* spp. than *Aspergillus* spp., suggesting that *Trichoderma* primarily drives VOC composition in co-cultures.

**FIGURE 2:**
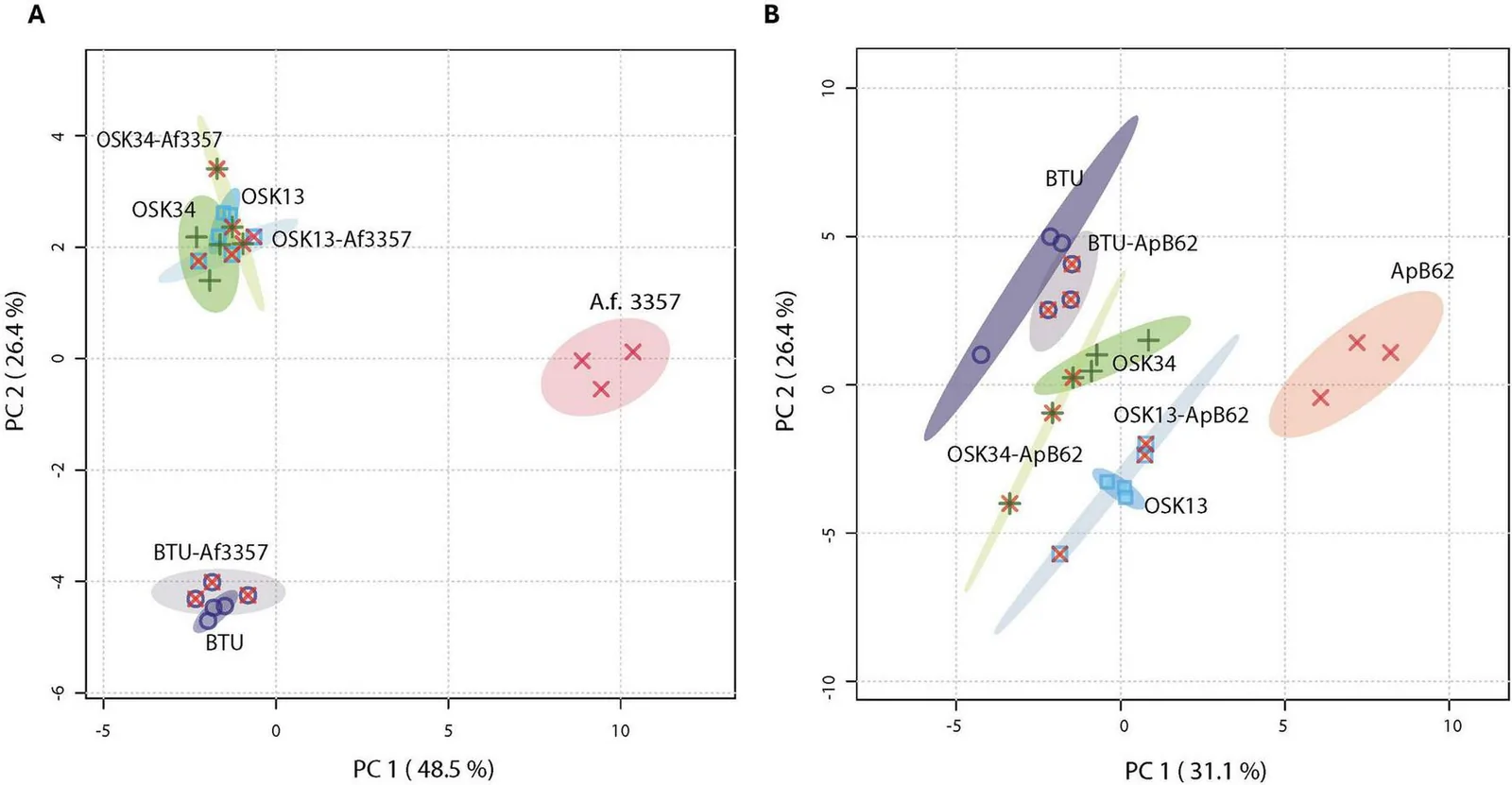
Principal Component Analysis (PCA) score plot of VOC profiles from *Trichoderma* and *Aspergillus* monocultures and their co-cultures with **(A)** *A. flavus* NRRL 3357 (A.f. 3357) and **(B)** *A. parasiticus B62* (ApB62). Each point represents an individual replicate with shaded ellipses indicating the 95% confidence region for each group (monocultures and co-cultures). Ellipses used to visualize the group separation and not statistical differences. OSK13: *T. virens*, OSK34: *T. harzianum*, and BTU: *T. asperellum*.

Among *Trichoderma* strains, OSK13 consistently grouped closer to OSK34 than to BTU, suggesting phylogenetic relatedness and VOC similarity between OSK13 and OSK34. In contrast, BTU exhibited a more distinct VOC profile, suggesting this species might employ a different inhibitory strategy with unique or amplified VOC biosynthesis pathways. *T. harzianum* (OSK34) and *T. virens* (OSK13) belong to a closely related lineage in the Harzianum/Virens clade, distinct from *T. asperellum* (BTU), which belongs to section *Trichoderma* (Kubicek et al., 2019). Species within the same phylogenetic clade tend to share more similar biosynthetic gene clusters, which likely translates to overlapping VOC production capabilities; however, each species also possesses unique biosynthetic gene clusters that likely contribute to the observed distinct VOC profiles (Li et al., 2021).

### 3.3 Shared and species-specific VOCs profiles in *Trichoderma*-*Aspergillus* interactions

A hierarchical clustered heatmap was generated to visualize overall VOC patterns and shifts from monocultures to co-cultures. The heatmap of VOC profiles revealed distinct clustering patterns among the three *Trichoderma* strains and their interactions with *Aspergillus* spp. (Figures 3A, B). Here, we focused on VOCs in three groups: (I) VOCs significantly higher in the *Trichoderma* strain with the most overall *Aspergillus* spp. inhibition (BTU; Voloshchuk et al., 2024), (II) VOCs significantly higher in *Trichoderma* strains and conserved across multiple species, and (III) VOCs shared by both *Aspergillus* spp. and *Trichoderma* spp. During antagonistic interactions, *Trichoderma* strains produced both strain-specific and shared VOCs (Figures 3A, B). VOCs significantly higher in BTU monoculture and co-cultures (*p* < 0.05) likely account for its distinct profile observed in the PCA and may contribute to its associated higher percent growth inhibition, which was significant for the A.f. 3357 co-culture (*p* < 0.05). Against A.f. 3357, three compounds were significantly higher in BTU (*p* < 0.05), both in monoculture and in co-culture, compared to all other *Trichoderma* interactions (Figures 3A, I). These metabolites have previously been reported for antifungal properties, such as 6-pentyl-2H-pyran-2-one (6PP), 2-Octanone, and 2-pentylfuran (Ismaiel and Ali, 2017; Wu et al., 2023; Thomas et al., 2025). 6PP is a well-characterized antifungal metabolite known to inhibit *A. flavus* growth and suppress aflatoxin B1 biosynthesis. Specifically, Ismaiel and Ali (2017) demonstrated that 6PP, at a concentration of 70 μg/mL, inhibited the growth of various *Aspergillus* spp. by up to 21% and reduced aflatoxin B1 production by 54%. In contrast, compounds such as 2-Octanone and 2-pentylfuran, along with others in this cluster, remain less studied as individual or synergistic antifungal agents, particularly against *Aspergillus* spp.

**FIGURE 3:**
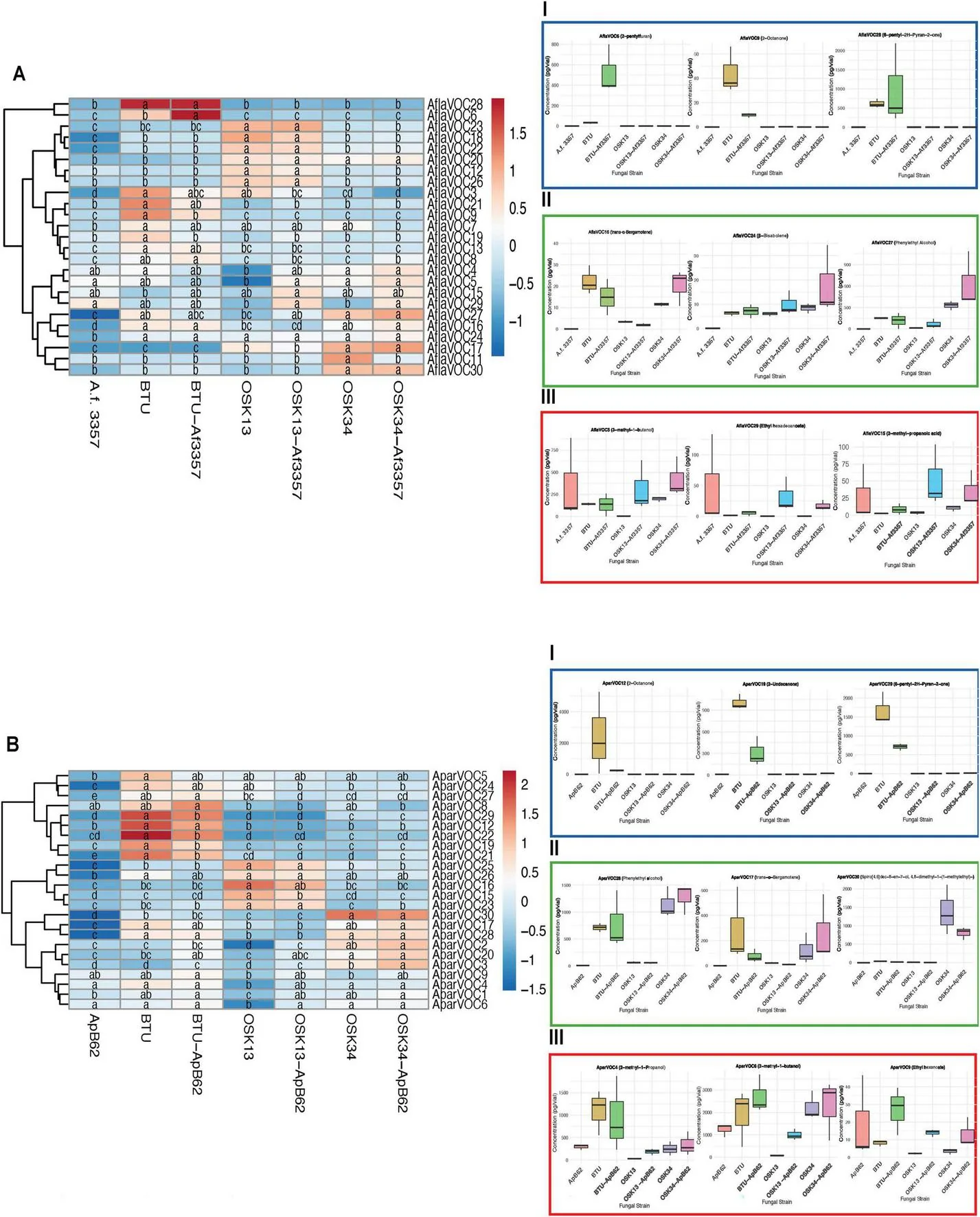
Heatmaps of VOC profiles from three *Trichoderma* strains and their interactions with **(A)** A.f. 3357 and **(B)** ApB62. Boxplots of example VOCs that are (I) significantly higher in BTU; (II) *Trichoderma* specific and shared across multiple species; and (III) produced by both *Aspergillus* and *Trichoderma.* Only VOCs that were significantly different by one-way ANOVA after FDR correction (adjusted *p* < 0.05) were visualized. Boxplot values represent the average of three biological replicates per treatment. Hierarchical clustering of VOC was conducted using Euclidean distance and complete linkage with a fixed sample order. For each VOC, treatments with a different letter are significantly different based on ANOVA followed by Tukey’s HSD *post hoc* testing (*p* < 0.05).

Some VOCs were shared across multiple *Trichoderma* spp. and were produced in significantly higher levels than in *Aspergillus*, though their responses in co-culture varied by species. Most of these VOCs belonged to compound classes previously reported to have antifungal properties, particularly sesquiterpenoids and alcohol derivatives (Boukaew et al., 2024; Stoppacher et al., 2010; Figure 3A, II). One example, phenylethyl alcohol, is produced by multiple *Trichoderma* spp. and has demonstrated efficacy in inhibiting *Aspergillus* spp. growth, sporulation, and aflatoxin production (Hua et al., 2014; Angel et al., 2016; Chan et al., 2023). Phenylethyl alcohol suppresses phytopathogens by disrupting cell membrane integrity and altering cellular processes. For instance, this compound induces reactive oxygen species stress against *Botrytis cinerea*, and causes downregulation of aflatoxin biosynthetic genes such as *aflR*, *pks* (polyketide synthase), and *nor-1* (norsolorinic acid reductase) in *A. flavus* (Hua et al., 2014; Zou et al., 2022). Phenylethyl alcohol concentration needed to completely inhibit AFB1 production is lower than the concentration needed to completely inhibit mycelial growth, meaning that they might have different mechanisms (Prakash et al., 2015). Teasing apart VOC mechanisms targeting toxin production versus growth could enable the development of biofungicides that reduce aflatoxin production without killing the fungus, which in turn could reduce the development of resistance. Other *Trichoderma*-specific VOCs, such as trans-α-bergamotene, β-bisabolene, and Spiro[4.5]dec-8-en-7-ol, 4,8-dimethyl-1-(1-methylethyl)- remain poorly characterized in *Trichoderma* spp. and have not been tested against *Aspergillus* spp., even though related bisabolene-types have shown antifungal activity against species like *Candida albicans* (Li et al., 2022; Bai et al., 2023; Yang et al., 2024). Future studies should validate these VOCs with a standard, test their antifungal activities and mechanisms against toxigenic *Aspergillus* spp., as they may represent a conserved VOC fingerprint underlying *Trichoderma* biocontrol activity across multiple species. This VOC fingerprint represents a starting point for studying conserved antifungal activities of VOCs produced by *Trichoderma* spp. against plant pathogens which could lead to the development of new biocontrol formulations.

The heatmap also highlights VOCs in the co-cultures that are produced by both *Aspergillus* and *Trichoderma* spp., with no significant differences between *Aspergillus* monocultures and the co-cultures (Figure 3A, III). These VOCs primarily include carboxylic acid/fatty acid esters such as ethyl hexadecanoate and short-chain alcohols such as 3-methyl-1-butanol. The production of these VOCs and others within the same chemical classes by multiple *Trichoderma* and *Aspergillus* spp. has been widely reported (Xu et al., 2014; Elsherbiny et al., 2020; Josselin et al., 2021; Elshahawy and Marrez, 2024). Interestingly, these compounds are consistently reported as some of the most abundant VOCs and key contributors to *Trichoderma* spp. antifungal activity when *Trichoderma* VOCs are profiled in monoculture (Elsherbiny et al., 2020; Elshahawy and Marrez, 2024). However, their inhibitory effects against toxigenic *Aspergillus* spp. appear complex. For example, it was reported that both 3-methyl-1-butanol and 2-methyl-1-butanol inhibited *A. flavus* growth by approximately 20% but increased aflatoxin B1 production by 1.5–2-fold (Zeringue and McCormick, 1990). This dual effect likely reflects the ecological versatility of these VOCs. Since these VOCs are produced by both the *Aspergillus* and the *Trichoderma* strain, their role in the co-culture as a defense, signaling, or antagonistic VOC depends on the timing and abundance produced by each fungus. This observation emphasizes the importance of comparing co-cultures to monocultures to better understand metabolite dynamics and their influence on interactions.

The clustered heatmap patterns and VOCs were mainly maintained across interactions with A.f. 3357 and ApB62 interactions, with a few differing VOCs (Figure 3B, I–III). This trend emphasizes the existence of both conserved and pathogen-specific VOC changes during the interactions of *Trichoderma* spp. and toxigenic *Aspergillus* spp. Differences in metabolite profiles among *Aspergillus* spp. may influence their VOC-mediated communication with *Trichoderma*, shaping the observed pathogen-specific interactions. The observed pathogen-specific variations could partly result from genetic mutations in strains such as ApB62, which are known to alter a variety of secondary metabolites (Roze et al., 2010). For both *Aspergillus* interactions, changes in VOC concentration from monoculture to co-culture also differed depending on the *Trichoderma* spp., reflecting adaptive modulation of antifungal metabolites and potentially explaining the difference in bioactivity across interactions.

### 3.4 Putative VOCs driving metabolomic shifts and informing selection of promising biocontrol strains in *Trichoderma*–*Aspergillus* interactions

We applied PLS-DA to identify putative VOCs discriminating between *Trichoderma* co-cultures. Alcohols, ketones, lactones, and terpenoids emerged as major discriminants, separating the different *Trichoderma-Aspergillus* co-cultures (Figures 4A, B). These VOC classes are well-documented for their production by *Trichoderma* with broad antifungal and anti-aflatoxigenic mechanisms of action, including disruption of membrane integrity, inhibition of spore germination, suppression of mycelial growth, and reduction of toxin biosynthesis (Almeida et al., 2023; Boukaew et al., 2024; Stoppacher et al., 2010). PLS-DA showed that most VOCs with high VIP scores were more abundant in BTU co-cultures compared to other *Trichoderma* strains. Specifically, four of six VOCs in the A.f. 3357 interaction and six of nine in the ApB62 interaction showed higher abundance in BTU, as indicated by deep red (Figures 4A, B). It is important to note that compounds with lower abundances can also play a role in bioactivity.

**FIGURE 4:**
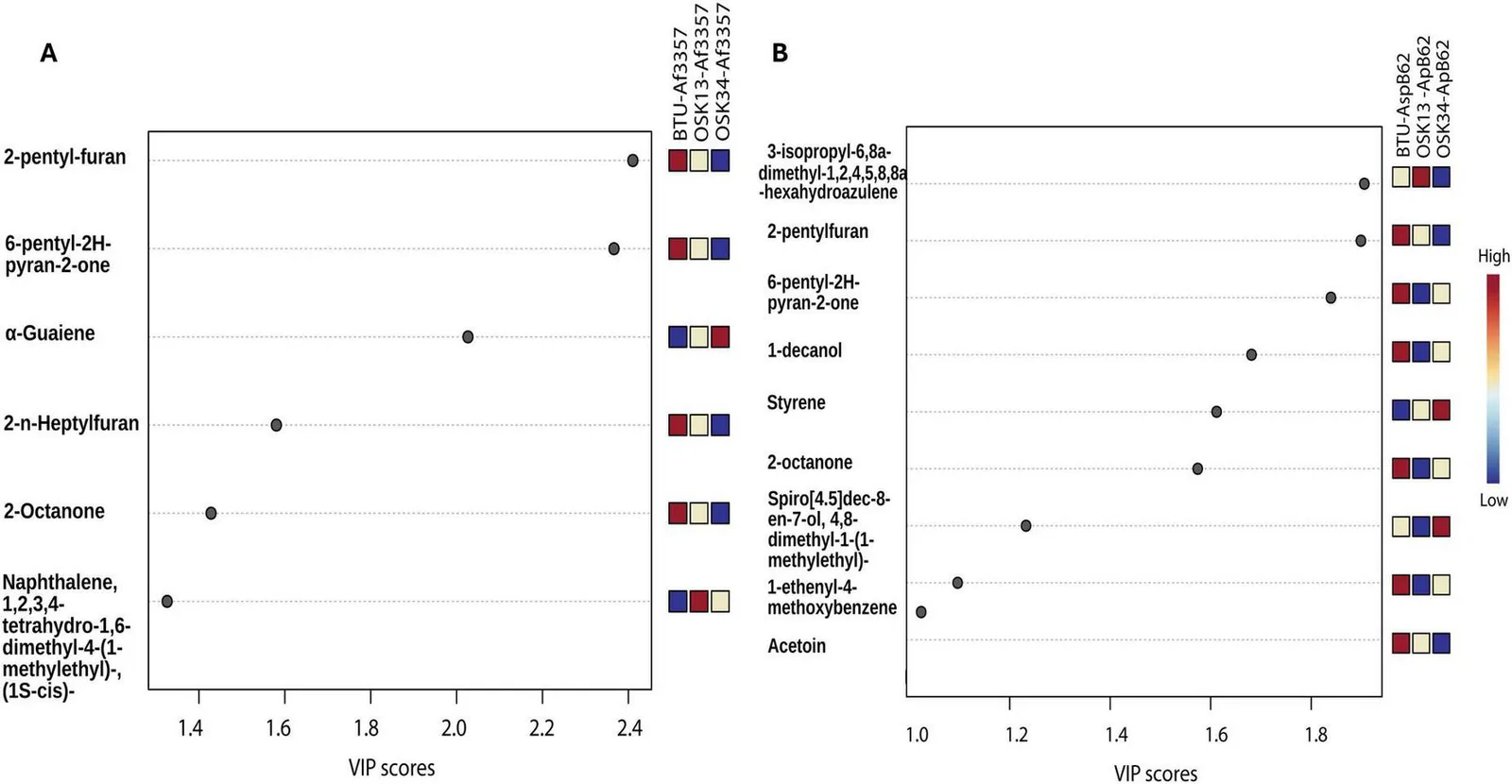
Variable importance in projection (VIP) score plots from PLS-DA showing VOCs with VIP score > 1 driving separation in the three *Trichoderma* interactions with **(A)** A.f. 3357 and **(B)** ApB62.

To identify putative antifungal VOCs, we supplemented PLS-DA results with statistical significance (ANOVA) and functional association (correlation with growth inhibition; Supplementary Figures 2A, B). This multi-criteria approach revealed four consensus VOCs that met all three criteria: (1) ranked high by PLS-DA (VIP > 1.0), (2) significantly different in co-cultures compared to their respective monocultures (ANOVA, *p* < 0.05), and (3) associated with antifungal activity (rs ≥ 0.5, *p* < 0.05). Notably, this approach identified four consensus VOCs: 2-pentylfuran, 1-decanol, 6-pentyl-2H-pyran-2-one, and 1-ethenyl-4-methoxybenzene, which were all predominantly enriched in BTU interactions with A.f. 3357 and ApB62 compared to all the other evaluated *Trichoderma* spp. (summarized in Supplementary Table 2). Amongst the four, 2-pentylfuran and 6-pentyl-2H-pyran-2-one have been confirmed with a standard, while 1-decanol and 1-ethenyl-4-methoxybenzene are putatively annotated with RI and MS, respectively.

Interestingly, these consensus VOCs exhibited distinct dynamics during co-culture. 2-pentylfuran was significantly upregulated in BTU co-culture with A.f. 3357 (*p* < 0.05), suggesting a potential upregulation of biosynthetic genes encoding this secondary metabolite during antagonism (Thomas et al., 2025). In contrast, the remaining three putative antifungal VOCs (1-decanol, 6-pentyl-2H-pyran-2-one, and 1-ethenyl-4-methoxybenzene) were significantly decreased during co-culture with ApB62 (*p* < 0.05), potentially reflecting biotransformation or active use in antibiosis during antagonistic interactions. These findings are consistent with previous reports showing that *Trichoderma* spp. alter their secondary metabolites in response to environmental cues and competing fungi (Nieto-Jacobo et al., 2017; Li et al., 2018; Gualtieri et al., 2022). Despite these dynamic changes, BTU maintained significantly higher abundances of all four compounds compared to other *Trichoderma* strains in both monoculture and co-culture conditions. This pattern suggests that initial high levels of VOC production, rather than dynamic upregulation alone, may also be a defining trait of superior biocontrol strains, ensuring sufficient bioactive concentrations are produced and maintained during fungal antagonism. While these putative antifungal VOCs have been reported in *Trichoderma* monocultures previously, this study is the first to identify them as discriminating compounds that distinguish strains associated with stronger inhibition of aflatoxin-producing *Aspergillus* strains, and to demonstrate how their abundance changes from monoculture to co-culture conditions within the context of a controlled experimental condition.

Overall, this work establishes a metabolomic framework for understanding VOC-mediated antagonism in *Trichoderma* and provides a volatile fingerprinting approach for selecting and optimizing strains with enhanced antifungal activity against aflatoxigenic *Aspergillus* spp. These findings provide initial insights for the development of metabolomics-informed biocontrol strategies for managing the growth of aflatoxin-producing fungi in food and agricultural systems. Some potential limitations of the study include that temporal fluctuations and compound degradation might have influenced the observed VOC concentrations, and the inability to definitely conclude the mechanism behind the final VOC changes in co-cultures. Hence, future studies integrating time-course VOC profiling with transcriptomic analysis could dissect the metabolite-induced mechanisms *Trichoderma* uses to inhibit the growth of toxigenic *Aspergillus* spp. Also, the use of deuterium-labeling to trace specific metabolites to their source organism in co-culture would help further parse out the contributions of *Trichoderma* vs. *Aspergillus* to VOC concentration changes in co-cultures.

In addition, while associations between VOC profiles and percent growth inhibition were observed, these 4 potential antifungal VOC findings remain exploratory due to the small sample-to-variable ratio of the data. Hence, independent and functional validation is still needed before any causal role is concluded. Future studies should include compound isolation, targeted bioassays, and transcriptomic analyses to confirm antifungal roles and elucidate specific molecular mechanisms. Finally, this study was conducted under laboratory conditions using nutrient-rich media, which may not reflect field performance. Evaluating how environmental factors and substrate variability affect VOC production will be essential to ensure reproducible biocontrol outcomes in real-world applications.

## 4 Conclusion

This study provides a comprehensive evaluation of volatile metabolomic shifts in *Trichoderma* spp. during interactions with *A. flavus* NRRL3357 and *A. parasiticus* B62, offering new insights into VOC-mediated biocontrol. Our findings reveal clear differences in VOC profiles among *Trichoderma* and *Aspergillus* monocultures and their co-cultures. Co-cultures displayed intermediate profiles closer to *Trichoderma*, supporting its dominant role in shaping the volatile composition during antagonism. Studying co-cultures offers deeper insights into potential mechanisms when compared to monocultures alone because co-cultures reveal specific VOC changes that are associated with the antagonistic interactions.

Metabolomic profiling underscored that antifungal efficacy is not driven by single compounds but by the collective action of multiple VOC classes, with both conserved and species-specific metabolites shaping bioactivity. Conserved compounds with previously noted antifungal activities, mainly terpenoids and alcohol, across *Trichoderma* spp. suggest a potential fingerprint of candidate VOCs driving the *Trichoderma* biocontrol strategy against toxigenic *Aspergillus*. *T. asperellum* (BTU) exhibited an enriched and distinct volatile profile, with elevated abundances of alcohols, ketones, lactones, and terpenoids compared to other strains. Several discriminant VOCs, including 2-pentylfuran, 1-decanol, 6-pentyl-2H-pyran-2-one, and 1-ethenyl-4-methoxybenzene, were consistently produced at higher levels by the *Trichoderma* strain that showed the strongest inhibition of toxigenic *A. flavus.* This association suggests that these compounds serve as candidate antifungal VOCs for identifying *Trichoderma* strains with enhanced biocontrol potential. Because aflatoxin production was not measured in the present study, the identified VOCs should be considered candidate biomarkers associated with *Aspergillus* growth inhibition rather than aflatoxin suppression.

## Data Availability

The datasets presented in this study can be found in online repositories. The names of the repository/repositories and accession number(s) can be found below: https://scholarsphere.psu.edu/resources/6cce6049-c4d5-4e40-9ff5-8ed9115d867a.

## Supplementary material

Supplementary Figure 1 Comparison of DVB/CAR/PDMS (gray, top two chromatograms) and PDMS/DVB (pink, bottom two chromatograms) fibers using raw chromatograms comparing the effect of fiber coating on volatile profiling of OSK13 using HS-SPME GC-MS. Two independent samples (1 and 2) were used to test each fiber. Peaks represent the relative abundance of compounds across retention time, and the arrows point to the retention time area of difference between the fibers. DVB, Divinylbenzene; CAR, Carboxen; PDMS, Polydimethylsiloxane.

Supplementary Figure 2 Spearman rank correlations between mean VOC abundance and percent growth inhibition of panel **(A**) A.f. 3357 and **(B)** ApB62. Semi-quantitative IS-normalized VOC concentrations were averaged across replicates of each *Trichoderma* spp. co-culture before analysis. Strong correlations were determined based on absolute correlation coefficients (r_s_ > 0.5 with *p* < 0.05).

## References

[B1] Agbetiameh D. Ortega-Beltran A. Awuah R. T. Atehnkeng J. Islam M.-S. Callicott K. A. et al. (2019). Potential of Atoxigenic *Aspergillus flavus* vegetative compatibility groups associated with maize and groundnut in ghana as biocontrol agents for Aflatoxin management. *Front. Microbiol.* 10:2069. 10.3389/fmicb.2019.02069 31555251 PMC6743268

[B2] Ali M. E. Gunn M. Stackhouse T. Waliullah S. Guo B. Culbreath A. et al. (2021). Sensitivity of *Aspergillus flavus* isolates from peanut seeds in georgia to Azoxystrobin, a Quinone outside Inhibitor (QoI) fungicide. *J. Fungi* 7:284. 10.3390/jof7040284 33918784 PMC8069585

[B3] Almeida O. A. C. de Araujo N. O. Dias B. H. S. de Sant’Anna Freitas C. Coerini L. F. Ryu C.-M. et al. (2023). The power of the smallest: The inhibitory activity of microbial volatile organic compounds against phytopathogens. *Front. Microbiol.* 13:951130. 10.3389/fmicb.2022.951130 36687575 PMC9845590

[B4] Angel L. P. L. Yusof M. T. Ismail I. S. Ping B. T. Y. Mohamed Azni I. N. A. Kamarudin N. H. et al. (2016). An in vitro study of the antifungal activity of *Trichoderma virens* 7b and a profile of its non-polar antifungal components released against *Ganoderma boninense*. *J. Microbiol.* 54, 732–744. 10.1007/s12275-016-6304-4 27796927

[B5] Atehnkeng J. Ojiambo P. S. Cotty P. J. Bandyopadhyay R. (2014). Field efficacy of a mixture of atoxigenic *Aspergillus flavus* Link:Fr vegetative compatibility groups in preventing aflatoxin contamination in maize (*Zea mays* L.). *Biol. Control* 72, 62–70. 10.1016/j.biocontrol.2014.02.009

[B6] Bai B. Liu C. Zhang C. He X. Wang H. Peng W. et al. (2023). *Trichoderma* species from plant and soil: An excellent resource for biosynthesis of terpenoids with versatile bioactivities. *J. Adv. Res.* 49, 81–102. 10.1016/j.jare.2022.09.010 36195283 PMC10334134

[B7] Boukaew S. Petlamul W. Yossan S. Srinuanpan S. Nooprom K. Zhang Z. (2024). Biofumigant potential and inhibition mechanism of *Trichoderma asperelloides* SKRU-01 volatile organic compounds for controlling aflatoxigenic *Aspergillus parasiticus* and *Aspergillus flavus* in stored peanuts. *Food Control* 157:110194. 10.1016/j.foodcont.2023.110194

[B8] Cardwell K. F. Henry S. H. (2004). Risk of Exposure to and mitigation of effect of aflatoxin on human health: A West African example. *J. Toxicol. Toxin Rev.* 23, 217–247. 10.1081/TXR-200027817

[B9] Chan M. E. Tan J. Y. Lee Y. Y. Lee D. Fong Y. K. Mutwil M. et al. (2023). Locally isolated *Trichoderma harzianum* species have broad spectrum biocontrol activities against the wood rot fungal species through both volatile inhibition and Mycoparasitism. *J. Fungi* 9:675. 10.3390/jof9060675 37367611 PMC10301622

[B10] Elshahawy I. E. Marrez D. A. (2024). Antagonistic activity of *Trichoderma asperellum* against *Fusarium* species, chemical profile and their efficacy for management of Fusarium-root rot disease in dry bean. *Pest Manag. Sci.* 80, 1153–1167. 10.1002/ps.7846 37874198

[B11] Elsherbiny E. A. Amin B. H. Aleem B. Kingsley K. L. Bennett J. W. (2020). *Trichoderma* volatile organic compounds as a biofumigation tool against late blight pathogen *Phytophthora infestans* in postharvest potato tubers. *J. Agric. Food Chem.* 68, 8163–8171. 10.1021/acs.jafc.0c03150 32790355

[B12] Eskola M. Kos G. Elliott C. T. Hajšlová J. Mayar S. Krska R. (2020). Worldwide contamination of food-crops with Mycotoxins: Validity of the widely cited ‘FAO estimate’ of 25%. *Crit. Rev. Food Sci. Nutr.* 60, 2773–2789. 10.1080/10408398.2019.1658570 31478403

[B13] Ferreira F. V. Musumeci M. A. (2021). *Trichoderma* as biological control agent: Scope and prospects to improve efficacy. *World J. Microbiol. Biotechnol.* 37:90. 10.1007/s11274-021-03058-7 33899136

[B14] Glare T. Caradus J. Gelernter W. Jackson T. Keyhani N. Köhl J. et al. (2012). Have biopesticides come of age? *Trends Biotechnol.* 30, 250–258. 10.1016/j.tibtech.2012.01.003 22336383

[B15] Gong Y. Y. Watson S. Routledge M. N. (2016). Aflatoxin exposure and associated human health effects, a review of epidemiological studies. *Food Saf.* 4, 14–27. 10.14252/foodsafetyfscj.2015026 32231900 PMC6989156

[B16] Gualtieri L. Monti M. M. Mele F. Russo A. Pedata P. A. Ruocco M. (2022). Volatile organic compound (VOC) profiles of different *Trichoderma* species and their potential application. *J. Fungi* 8:989. 10.3390/jof8100989 36294554 PMC9605199

[B17] Guzmán-Guzmán P. Kumar A. de los Santos-Villalobos S. Parra-Cota F. I. Orozco-Mosqueda M. C. Fadiji A. E. et al. (2023). *Trichoderma* species: Our best fungal allies in the biocontrol of plant diseases—a review. *Plants* 12:432. 10.3390/plants12030432 36771517 PMC9921048

[B18] Hu J. Zhou Y. Chen K. Li J. Wei Y. Wang Y. et al. (2020). Large-scale *Trichoderma* diversity was associated with ecosystem, climate and geographic location. *Environ. Microbiol.* 22, 1011–1024. 10.1111/1462-2920.14798 31498946

[B19] Hua S. S. T. Beck J. J. Sarreal S. B. L. Gee W. (2014). The major volatile compound 2-phenylethanol from the biocontrol yeast, *Pichia anomala*, inhibits growth and expression of Aflatoxin biosynthetic genes of *Aspergillus flavus*. *Mycotoxin Res.* 30, 71–78. 10.1007/s12550-014-0189-z 24504634

[B20] Irakoze Z. Wee J. Hopfer H. Voloshchuk N. Lambert J. (2026). *Comprehensive Datasets for Signaling in the Gas Phase: Comparative Volatile Profiling of Biocontrol Trichoderma spp. and Toxigenic Aspergillus spp. in Isolation and Co-culture using HS-SPME GC-MS*, Scholarsphere. 10.26207/bj6m-8y43

[B21] Ismaiel A. A. Ali D. M. I. (2017). Antimicrobial properties of 6-pentyl-α-pyrone produced by endophytic strains of *Trichoderma koningii* and its effect on aflatoxin B1 production. *Biologia* 72, 1403–1415. 10.1515/biolog-2017-0173

[B22] Johnsen L. G. Skou P. B. Khakimov B. Bro R. (2017). Gas chromatography – mass spectrometry data processing made easy. *J. Chromatogr. A* 1503, 57–64. 10.1016/j.chroma.2017.04.052 28499599

[B23] Josselin L. De Clerck C. De Boevre M. Moretti A. Jijakli M. H. Soyeurt H. et al. (2021). Volatile organic compounds emitted by *Aspergillus flavus* strains producing or not Aflatoxin B1. *Toxins* 13:705. 10.3390/toxins13100705 34678998 PMC8539470

[B24] Kew M. C. (2003). Synergistic interaction between aflatoxin B1 and hepatitis B virus in hepatocarcinogenesis. *Liver Int.* 23, 405–409. 10.1111/j.1478-3231.2003.00869.x 14986813

[B25] Kifle M. H. Yobo K. S. Laing M. D. (2017). Biocontrol of *Aspergillus flavus* in groundnut using *Trichoderma harzianum* stain kd. *J. Plant. Dis. Prot.* 124, 51–56. 10.1007/s41348-016-0066-4

[B26] Kos J. Anić M. Radić B. Zadravec M. Janić Hajnal E. Pleadin J. (2023). Climate change—A global threat resulting in increasing Mycotoxin occurrence. *Foods* 12:2704. 10.3390/foods12142704 37509796 PMC10379110

[B27] Kubicek C. P. Steindorff A. S. Chenthamara K. Manganiello G. Henrissat B. Zhang J. et al. (2019). Evolution and comparative genomics of the most common *Trichoderma* species. *BMC Genomics* 20:485. 10.1186/s12864-019-5680-7 31189469 PMC6560777

[B28] Leggieri M. C. Toscano P. Battilani P. (2021). Predicted Aflatoxin B1 increase in europe due to climate change: Actions and reactions at global level. *Toxins* 13:292. 10.3390/toxins13040292 33924246 PMC8074758

[B29] Lewis M. H. Carbone I. Luis J. M. Payne G. A. Bowen K. L. Hagan A. K. et al. (2019). Biocontrol strains differentially shift the genetic structure of indigenous soil populations of *Aspergillus flavus*. *Front. Microbiol.* 10:1738. 10.3389/fmicb.2019.01738 31417528 PMC6685141

[B30] Li H.-Y. Yang W.-Q. Zhou X.-Z. Shao F. Shen T. Guan H.-Y. et al. (2022). Antibacterial and antifungal Sesquiterpenoids: Chemistry, resource, and activity. *Biomolecules* 12:1271. 10.3390/biom12091271 36139108 PMC9496053

[B31] Li N. Alfiky A. Wang W. Islam M. Nourollahi K. Liu X. et al. (2018). Volatile compound-mediated recognition and inhibition between *Trichoderma* biocontrol agents and *Fusarium oxysporum*. *Front. Microbiol.* 9:2614. 10.3389/fmicb.2018.02614 30455673 PMC6231246

[B32] Li W.-C. Lin T.-C. Chen C.-L. Liu H.-C. Lin H.-N. Chao J.-L. et al. (2021). Complete genome sequences and genome-wide characterization of *Trichoderma* biocontrol agents provide new insights into their evolution and variation in genome organization, sexual development, and fungal-plant interactions. *Microbiol. Spectr.* 9:e00663-21. 10.1128/Spectrum.00663-21 34908505 PMC8672877

[B33] Mishra N. Khan S. S. Sundari S. K. (2016). Native isolate of *Trichoderma*: A biocontrol agent with unique stress tolerance properties. *World J. Microbiol. Biotechnol.* 32:130. 10.1007/s11274-016-2086-4 27339311

[B34] Mostafa A. A. Al-Rahmah A. Abdel-Megeed A. Sayed S. Hatamleh A. (2013). Antagonistic activities of some fungal strains against the toxigenic *Aspergillus flavus* Isolate and its Aflatoxins productivity. *J. Pure Appl. Microbiol.* 7, 169–178.

[B35] National Institute of Standards and Technology [NIST]. (2014). *NIST Standard Reference Database 1A.* Available online at: https://www.nist.gov/srd/nist-standard-reference-database-1a (accessed November 13, 2025).

[B36] Nieto-Jacobo M. F. Steyaert J. M. Salazar-Badillo F. B. Nguyen D. V. Rostás M. Braithwaite M. et al. (2017). Environmental growth conditions of *Trichoderma* spp. affects indole acetic acid derivatives, volatile organic compounds, and plant growth promotion. *Front. Plant Sci.* 8:102. 10.3389/fpls.2017.00102 28232840 PMC5299017

[B37] Pang Z. Lu Y. Zhou G. Hui F. Xu L. Viau C. et al. (2024). MetaboAnalyst 6.0: Towards a unified platform for metabolomics data processing, analysis and interpretation. *Nucleic Acids Res.* 52, W398–W406. 10.1093/nar/gkae253 38587201 PMC11223798

[B38] Peles F. Sipos P. Kovács S. Gyõri Z. Pócsi I. Pusztahelyi T. (2021). Biological control and mitigation of aflatoxin contamination in commodities. *Toxins* 13:104. 10.3390/toxins13020104 33535580 PMC7912779

[B39] Posit team,. (2024). *RStudio: Integrated Development Environment for R.* Available online at: http://www.posit.co/ (accessed May 11, 2025).

[B40] Prakash B. Singh P. Goni R. Raina A. K. P. Dubey N. K. (2015). Efficacy of Angelica archangelica essential oil, phenyl ethyl alcohol and α- terpineol against isolated molds from walnut and their antiaflatoxigenic and antioxidant activity. *J. Food Sci. Technol.* 52, 2220–2228. 10.1007/s13197-014-1278-x PMC4375230 25829603

[B41] Probst C. Bandyopadhyay R. Price L. E. Cotty P. J. (2011). Identification of atoxigenic *Aspergillus flavus* isolates to reduce aflatoxin contamination of maize in Kenya. *Plant Dis.* 95, 212–218. 10.1094/PDIS-06-10-0438 30743416

[B42] Ren X. Branà M. T. Haidukowski M. Gallo A. Zhang Q. Logrieco A. F. et al. (2022). Potential of *Trichoderma* spp. for biocontrol of aflatoxin-producing *Aspergillus flavus*. *Toxins* 14:86. 10.3390/toxins14020086 35202114 PMC8875375

[B43] Ren X. Zhang Q. Zhang W. Mao J. Li P. (2020). Control of Aflatoxigenic molds by antagonistic microorganisms: Inhibitory behaviors, bioactive compounds, related mechanisms, and influencing factors. *Toxins* 12:24. 10.3390/toxins12010024 31906282 PMC7020460

[B44] Roze L. V. Chanda A. Laivenieks M. Beaudry R. M. Artymovich K. A. Koptina A. V. et al. (2010). Volatile profiling reveals intracellular metabolic changes in *Aspergillus parasiticus*: veA regulates branched chain amino acid and ethanol metabolism. *BMC Biochem.* 11:33. 10.1186/1471-2091-11-33 20735852 PMC2939540

[B45] Ruangwong O.-U. Wonglom P. Suwannarach N. Kumla J. Thaochan N. Chomnunti P. et al. (2021). Volatile organic compound from *Trichoderma asperelloides* TSU1: Impact on plant pathogenic fungi. *J. Fungi* 7:187. 10.3390/jof7030187 33807949 PMC7999943

[B46] Shahiri T. M. Rahnama K. Jahanshahi M. Nasrollahnejad S. Fatemi M. H. (2016). Identification of volatile organic compounds of some *Trichoderma* species using static headspace gas chromatography-mass spectrometry. *MI* 3, 47–55. 10.22043/mi.2017.41532.1072

[B47] Sivparsad B. J. Laing M. D. (2016). Pre-harvest silk treatment with *Trichoderma harzianum* reduces aflatoxin contamination in sweetcorn. *J. Plant Dis. Prot.* 123, 285–293. 10.1007/s41348-016-0037-9

[B48] Stoppacher N. Kluger B. Zeilinger S. Krska R. Schuhmacher R. (2010). Identification and profiling of volatile metabolites of the biocontrol fungus *Trichoderma atroviride* by HS-SPME-GC-MS. *J. Microbiol. Methods* 81, 187–193. 10.1016/j.mimet.2010.03.011 20302890

[B49] Thomas G. A. Vuts J. Withall D. M. Caulfield J. C. Sidda J. Grant M. R. et al. (2025). Inducible volatile chemical signalling drives antifungal activity of *Trichoderma hamatum* GD12 during confrontation with the pathogen *Sclerotinia sclerotiorum*. *Environ. Microbiol. Rep.* 17:e70192. 10.1111/1758-2229.70192 40996658 PMC12462542

[B50] Tsitsigiannis D. I. Dimakopoulou M. Antoniou P. P. Tjamos E. C. (2012). Biological control strategies of mycotoxigenic fungi and associated mycotoxins in Mediterranean basin crops. *Phytopathol. Mediterranea* 51, 158–174.

[B51] United Nations. (2023). *Population.* Manhattan, NJ: United Nations.

[B52] van Zijll de Jong E. Kandula J. Rostás M. Kandula D. Hampton J. et al. (2023). Fungistatic activity mediated by volatile organic compounds is isolate-dependent in *Trichoderma* sp. “atroviride B.”. *J. Fungi* 9:238. 10.3390/jof9020238 36836354 PMC9965825

[B53] Voloshchuk N. Irakoze Z. Kang S. Kellogg J. Wee J. (2024). Three ecological models to evaluate the effectiveness of *Trichoderma* spp. for suppressing Aflatoxigenic *Aspergillus flavus* and *Aspergillus parasiticus*. *Toxins* 16:314. 10.3390/toxins16070314 39057954 PMC11281256

[B54] Weaver M. A. Callicott K. A. Mehl H. L. Opoku J. Park L. C. Fields K. S. et al. (2022). Characterization of the *Aspergillus flavus* population from highly aflatoxin-contaminated corn in the United States. *Toxins* 14:755. 10.3390/toxins14110755 36356005 PMC9698142

[B55] Wee J. Day D. M. Linz J. E. (2016). Effects of zinc chelators on aflatoxin production in *Aspergillus parasiticus*. *Toxins* 8:171. 10.3390/toxins8060171 27271668 PMC4926138

[B56] Wu Y. Li X. Dong L. Liu T. Tang Z. Lin R. et al. (2023). A new insight into 6-Pentyl-2H-pyran-2-one against *Peronophythora litchii* via TOR pathway. *J. Fungi* 9:863. 10.3390/jof9080863 37623635 PMC10515317

[B57] Xu J. Luo X. Zhong W. Zhang J. Tan R. (2014). Characterization of volatile constituents from an endophytic *Aspergillus fumigatus* strain. *J. Chem. Pharm. Res.* 6, 893–897.

[B58] Yalage Don S. M. Schmidtke L. M. Gambetta J. M. Steel C. C. (2020). *Aureobasidium pullulans* volatilome identified by a novel, quantitative approach employing SPME-GC-MS, suppressed *Botrytis cinerea* and *Alternaria alternata* in vitro. *Sci. Rep.* 10:4498. 10.1038/s41598-020-61471-8 32161291 PMC7066187

[B59] Yang W. Tian S. Du Y.-F. Zeng X.-L. Liang J.-J. Lan W.-J. et al. (2024). Genome mining of the marine-derived fungus *Trichoderma erinaceum* F1-1 unearths bergamotene-type sesquiterpenoids. *J. Nat. Prod.* 87, 2746–2756. 10.1021/acs.jnatprod.4c00905 39623525

[B60] Yu J. Hennessy D. A. Tack J. Wu F. (2022). Climate change will increase aflatoxin presence in US Corn. *Environ. Res. Lett.* 17:054017. 10.1088/1748-9326/ac6435

[B61] Zeringue H. J. McCormick S. P. (1990). Aflatoxin production in cultures of *Aspergillus flavus* incubated in atmospheres containing selected cotton leaf-derived volatiles. *Toxicon* 28, 445–448. 10.1016/0041-0101(90)90083-J 2112274

[B62] Zou X. Wei Y. Jiang S. Xu F. Wang H. Zhan P. et al. (2022). ROS stress and cell membrane disruption are the main antifungal mechanisms of 2-phenylethanol against *Botrytis cinerea*. *J. Agric. Food Chem.* 70, 14468–14479. 10.1021/acs.jafc.2c06187 36322824

