# Supplementary material for: Signaling in the gas phase: comparative volatile profiling of biocontrol Trichoderma spp. and aflatoxin-producing Aspergillus spp. in isolation and co-culture using HS-SPME GC-MS
Source: Front Microbiol. 2026 Oct 9;17:1873321. doi: 10.3389/fmicb.2026.1873321 (PMC13690720; doi:10.3389/fmicb.2026.1873321)
Supplement: Supplementary file 1 [file Image_1.pdf]

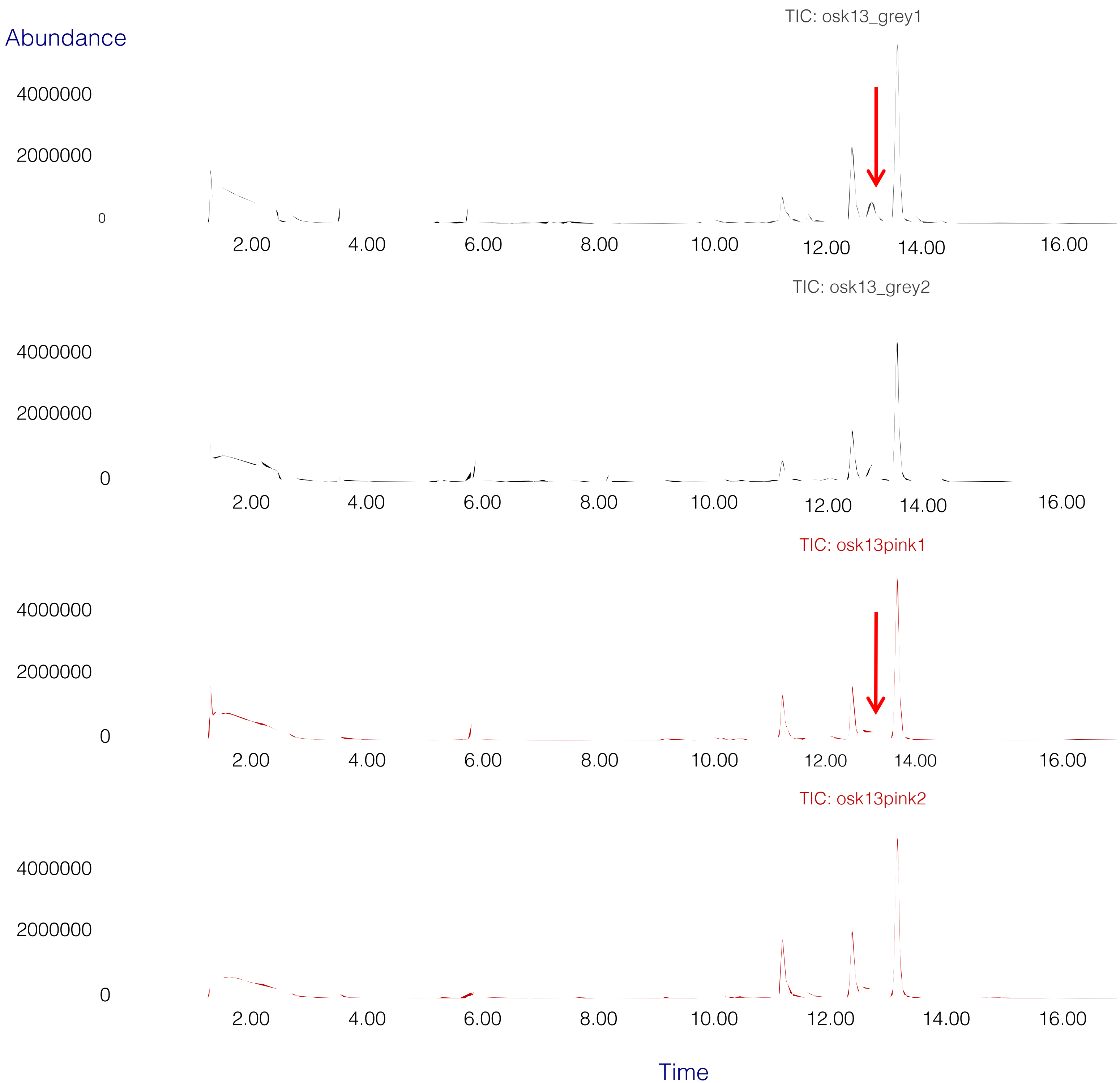

Abundance
TIC: osk13_grey1
4000000
2000000
0
2.00
4.00
6.00
8.00
10.00
12.00
14.00
16.00
TIC: osk13_grey2
4000000
2000000
0
2.00
4.00
6.00
8.00
10.00
12.00
14.00
16.00
TIC: osk13pink1
4000000
2000000
0
2.00
4.00
6.00
8.00
10.00
12.00
14.00
16.00
TIC: osk13pink2
4000000
2000000
0
2.00
4.00
6.00
8.00
10.00
12.00
14.00
16.00
Time
