# Supplementary material for: Signaling in the gas phase: comparative volatile profiling of biocontrol Trichoderma spp. and aflatoxin-producing Aspergillus spp. in isolation and co-culture using HS-SPME GC-MS
Source: Front Microbiol. 2026 Oct 9;17:1873321. doi: 10.3389/fmicb.2026.1873321 (PMC13690720; doi:10.3389/fmicb.2026.1873321)
Supplement: Supplementary file 2 [file Image_2.pdf]

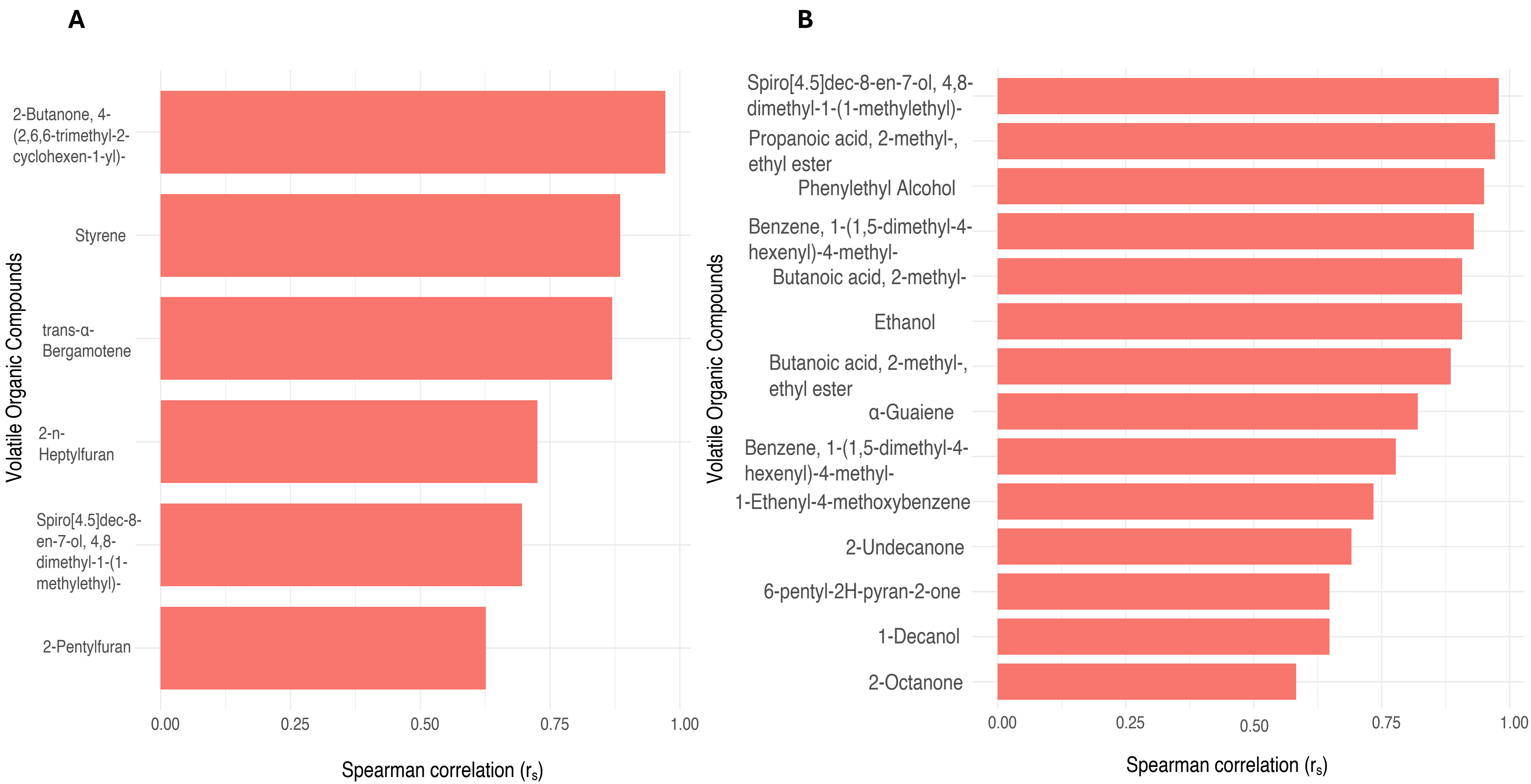
A
2-Butanone, 4-(2,6,6-trimethyl-2-cyclohexen-1-yl)-
Styrene
trans-α-Bergamotene
2-n-Heptylfuran
Spiro[4.5]dec-8-en-7-ol, 4,8-dimethyl-1-(1-methylethyl)-
2-Pentylfuran
Volatile Organic Compounds
0.00
0.25
0.50
0.75
1.00
Spearman correlation (r_s)
B
Spiro[4.5]dec-8-en-7-ol, 4,8-dimethyl-1-(1-methylethyl)-
Propanoic acid, 2-methyl-, ethyl ester
Phenylethyl Alcohol
Benzene, 1-(1,5-dimethyl-4-hexenyl)-4-methyl-
Butanoic acid, 2-methyl-
Ethanol
Butanoic acid, 2-methyl-, ethyl ester
α-Guaiene
Benzene, 1-(1,5-dimethyl-4-hexenyl)-4-methyl-
1-Ethenyl-4-methoxybenzene
2-Undecanone
6-pentyl-2H-pyran-2-one
1-Decanol
2-Octanone
Volatile Organic Compounds
0.00
0.25
0.50
0.75
1.00
Spearman correlation (r_s)
