# Supplementary material for: Signaling in the gas phase: comparative volatile profiling of biocontrol Trichoderma spp. and aflatoxin-producing Aspergillus spp. in isolation and co-culture using HS-SPME GC-MS
Source: Front Microbiol. 2026 Oct 9;17:1873321. doi: 10.3389/fmicb.2026.1873321 (PMC13690720; doi:10.3389/fmicb.2026.1873321)
Supplement: Supplementary file 3 [file Table_1.docx]

**Table S1** List of pure analytical standards

| **Compound Name (>98% purity)** | **CAS#** | **Supplier** |
| --- | --- | --- |
| Propanoic acid, 2-methyl-, ethyl ester | 97-62-1 | Tokyo Chemical Industries (TCI America) |
| Butanoic acid, 2-methyl-, ethyl ester | 7452-79-1 | Tokyo Chemical Industries (TCI America) |
| 2-methyl-1-propanol | 78-83-1 | Sigma-Aldrich Fine Chemicals |
| 2-methyl-1-butanol | 137-32-6 | Thermo Fisher Scientific (Alfa Aesar) |
| 3-methyl-1-butanol | 123-51-3 | Sigma Aldrich |
| 2-pentylfuran | 3777-69-3 | Thermo Fisher Scientific (Alfa Aesar) |
| Ethyl hexanoate | 123-66-0 | Sigma-Aldrich Fine Chemicals |
| 3-Octanone | 106-68-3 | Tokyo Chemical Industries (TCI America) |
| 2-Octanone | 111-13-7 | Tokyo Chemical Industries (TCI America) |
| Acetoin | 513-86-0 | Sigma-Aldrich Fine Chemicals |
| Butanoic acid, 2-methyl- | 116-53-0 | Thermo Fisher Scientific (Alfa Aesar) |
| Phenylethyl Alcohol | 60-12-8 | Sigma-Aldrich Fine Chemicals |
| 6-pentyl-2H-pyran-2-one | 27593-23-3 | Sigma-Aldrich |
| Acetic acid | 64-19-7 | Sigma-Aldrich |
| Caryophyllene | 87-44-5 | Sigma-Aldrich |
| Ethyl hexadecanoate | 628-97-7 | Sigma-Aldrich |

**Table S2**. Summary of Candidate antifungal VOCs of *Trichoderma* spp. against *Aspergillus* spp.

| VOC | Identification Method | Enriched In | Statistical Evidence | Relationship to Growth Inhibition | Level of Evidence | Notes |
| --- | --- | --- | --- | --- | --- | --- |
| 6-Pentyl-2H-pyran-2-one (6PP) | MS + Analytical Standard | BTU monoculture and BTU co-cultures | VIP > 1;  ANOVA p < 0.05;  Spearman rs ≥ 0.5 | Positive association | High | Previously reported antifungal compound; confirmed with analytical standard |
| 2-Pentylfuran | MS + RI + Analytical Standard | BTU monoculture and BTU co-cultures | VIP > 1;  ANOVA p < 0.05; Spearman rs ≥ 0.5 | Positive association | High | Significantly upregulated in BTU–*A. flavus* co-culture |
| 1-Decanol | RI | BTU monoculture and BTU co-cultures | VIP > 1;  ANOVA p < 0.05; Spearman rs ≥ 0.5 | Positive association | Moderate | Tentatively identified by RI; requires functional validation |
| 1-Ethenyl-4-methoxybenzene | MS | BTU monoculture and BTU co-cultures | VIP > 1;  ANOVA p < 0.05; Spearman rs ≥ 0.5 | Positive association | Moderate | Tentatively identified by MS only |
